# ExAgBov: A public database of annotated variations from hundreds of bovine whole-exome sequencing samples

**DOI:** 10.1038/s41597-022-01597-8

**Published:** 2022-08-02

**Authors:** Rotem Raz, Zvi Roth, Moran Gershoni

**Affiliations:** 1 Department of Ruminant Science, Institute of Animal Sciences, Agricultural Research Organization, The Volcani Center, Rishon LeZion, 7505101 Israel; 2 Department of Animal Sciences, Robert H. Smith Faculty of Agriculture, Food and Environment, the Hebrew University, Rehovot, 76100 Israel

**Keywords:** Agricultural genetics, Animal breeding

## Abstract

Large reference datasets of annotated genetic variations from genome-scale sequencing are essential for interpreting identified variants, their functional impact, and their possible contribution to diseases and traits. However, to date, no such database of annotated variation from broad cattle populations is publicly available. To overcome this gap and advance bovine NGS-driven variant discovery and interpretation, we obtained and analyzed raw data deposited in the SRA public repository. Short reads from 262 whole-exome sequencing samples of *Bos Taurus* were mapped to the *Bos Taurus* ARS-UCD1.2 reference genome. The GATK best practice workflow was applied for variant calling. Comprehensive annotation of all recorded variants was done using the Ensembl Variant Effect Predictor (VEP). An in-depth analysis of the population structure revealed the breeds comprising the database. The Exomes Aggregate of Bovine- ExAgBov is a comprehensively annotated dataset of more than 20 million short variants, of which ~2% are located within open reading frames, splice regions, and UTRs, and more than 60,000 variants are predicted to be deleterious.

## Abstract (summary)

**Table:** 

Measurement(s)	Genetic variation
Technology Type(s)	Whole Exome Sequencing
Factor Type(s)	Diversity • Coverage • Population Structure
Sample Characteristic - Organism	Bos taurus

## Background & Summary

Genetic variation databases are essential for the study of species population and biology^1–3^ and, particularly, for interpreting variants in the context of diseases or other phenotypes^4^. For instance, a key step in discovering causal variation is filtering candidate variants by their frequency. In the last decade, several databases for human genetic variation, such as the 1000 Genomes Project^5^, the Exome Variant Server^1^, and the gnomAD^6^, have become publicly available. However, in animal studies such resources are sparse or altogether absent, specifically for livestock that is intensively studied for genetic and genomic selection^7^.

About a decade ago, the 1000 Bull Genomes Project was initiated to facilitate accurate imputation of animals genotyped with SNP arrays using whole-genome sequence data to find causative mutations affecting economic and health traits in beef and dairy cattle^2^. As of 2019, this project reported whole-genome sequences of 2,703 individuals representing a large proportion of the world’s cattle diversity. Analysis of these data yielded more than 80 million single-nucleotide variations (SNVs) and small insertion deletions (INDELs), which has contributed to the identification of deleterious mutations associated with diseases and embryonic lethality^2,3^. However, although the per-chromosome VCF files for 1832 samples are available at the European Nucleotide Archive website^8^ to our knowledge, the entire database of annotated variation including their population allele frequencies, is not publicly available. Moreover, the requirements for joining the consortium are not feasible for every research group.

To address this need and improve genome-scale sequencing-driven research, we created a genetic variation database of cattle from short reads deposited in public databases. As of June 2020, we have obtained all available bovine whole-exome sequencing (WES) samples deposited at the NCBI Sequences Reads Archive (SRA) database. By querying the SRA identifiers, all bovine WES samples were identified, and their fastq files were downloaded. We then mapped the fastq short reads to the most updated *Bos Taurus* reference genome (ARS-UCD1.2)^9^ and conducted variant calling by applying the GATK best practice recommended workflow^10^. This step produced a joint Variant Call Format (VCF) file of all the 262 samples. The joint VCF file includes the genotypes of all individuals, allele number (AN), allele frequency (AF), the GATK inbreeding coefficient, and the coverage depth (DP) of each variant. The inbreeding coefficient measures the excess heterozygosity at a variant site. A very negative value can be used as a proxy for mapping quality, whereas positive values can account for biases due to population stratification or inbreeding. We revealed the main breeds that compose the database by population structure analysis. Using kinship analysis, we found that only a few samples are closely related, and most of the bovine samples share only a small fraction of their genomes. The current release of the ExAgBov database records more than 20 million short variants, similar to the first reports from the 1000 Bull Genomes Project, which was carried out on whole-genome sequencing (WGS) samples from 234 animals^3^. This dataset is a useful tool for variant annotation in WGS and WES studies. In addition, it can be used to search for variations around quantitative trait loci identified in genome-wide association studies or any genes of interest.

## Methods

### Data acquisition and short read mapping

We used the SRA Toolkit (https://trace.ncbi.nlm.nih.gov/Traces/sra/sra.cgi?view=software) to obtain from the publicly available SRA at NCBI (https://www.ncbi.nlm.nih.gov/sra) fastq files of bovine short reads from 262 *Bos Taurus* samples. A metadata file of all samples was deposited in the ExAgBov repository at Mendeley 10.17632/m3p9m9vc4g.3. Exome sequences were analyzed as follows: Raw reads were mapped to the reference genome (*Bos taurus* ARS-UCD1.2)^9^ downloaded from the Ensembl genome browser (http://ftp.ensembl.org/pub/release-104/fasta/bos_taurus/dna/). Using the default arguments, reads were aligned to this genome build by the Burrows-Wheeler Aligner software (bwa-mem algorithm)^11^. Next, to avoid biases introduced by data generation steps such as PCR amplification, PCR duplicates were removed using Picard tool (version 2.20.2; https://broadinstitute.github.io/picard/). Base quality score recalibration (BQSR) was performed to detect errors made by the sequencing machine, and the curated BAM files were coordinated, sorted, and indexed using the Picard algorithm.

### Variant calling and annotation

Variant calling was carried out with the Genome Analysis Tool Kit (GATK, version 4.1.6.0)^10^ as recommended by the GATK workflow. SNVs and INDELs variants were called via local re-assembly of haplotypes that were generated by the HaplotypeCaller algorithm. The HaplotypeCaller was run per sample to generate an intermediate genomic VCF (g.VCF) file, which was then used for efficient joint genotyping of multiple samples while retaining coverage data for uncalled or non-polymorphic sites. Genotype files were produced with the CombineGVCFs algorithm by first combining the g.VCF files of all samples and then by a joint call of the combined g.VCF file. The joint call procedure allows the calculation of the GATK inbreeding coefficient for each variant. This measurement, which is based on the Hardy-Weinberg principle, was calculated as: 1- [observed heterozygotes / expected heterozygotes]. Finally, all variants in the combined VCF file underwent comprehensive annotation by the Ensembl VEP^12^. Because the analyzed samples were possibly obtained from different sequencing platforms using different read lengths and coverages, we calculated and obtained the median, average, and maximal sequence depth for all documented variants. In addition, the DP of all variants in all samples is provided in the ExAgBov repository^13^. Variant calling in the complete database was carried out without filtering, allowing users to use the supporting information and perform filtering steps according to their requirements and needs. In addition, we produce a filtered database by performing the following filtering steps: we calculated the 95^th^ percentile depth of coverage for each of the variants and then filtered out only those variants that passed 95^th^ percentile >  = 4 with a mean quality (MQ) > 30. The filtering steps retained > 2.7 million variants, and these variants’ consequence distribution can be found in figure S1. The outcome filtered database file is also provided in the ExAgBov repository^13^, at https://data.mendeley.com/datasets/m3p9m9vc4g/.

### ExAgBov population structure analysis

To assess the population structure of the ExAgBov database and relatedness among samples, we computed a kinship matrix. From the joint VCF file, we obtained more than 860 thousand informative alleles with a total number of alleles called (AN) > 150, allele frequency (AF) > 0.03, and mean quality (MQ) > 30. Next, using plink software^14^, we transformed the filtered VCF into plink bed and ped files using the make-bed flag. The identical by state (IBS) similarity matrix was then calculated using the --genome flag and a multidimensional scaling (MDS) analysis was carried out with --cluster and --mds_plot arguments. Then, using KING software^15^, we computed the pairwise kinship levels among all ExAgBov samples by calculating the proportion of Identical By Descent (IBD) genomic segments shared by each bovine pair. The resulting table was then converted to a kinship matrix by a Perl script, where the columns and rows are the bovine WES samples, and the bins are the IBD proportion of each pair. The kinship matrix was hierarchically clustered by the Pearson correlation coefficient using Morpheus software (https://software.broadinstitute.org/Morpheus) and the population structure was visualized as a clustered heatmap.

To assess the degree of inbreeding in the population, we calculated the Runs of Homozygosity (ROH)^15^ using the KING software. The inferred breed affiliation of the clustered samples in the MDS and the hierarchically clustered matrix was determined by obtaining the WES sample breeds from the SRA metadata (see 10.17632/m3p9m9vc4g.3^13^), whenever denoted in the relevant SRA project. Thus, unknown samples within a cluster of known samples were assumed to be from the same or closely related breed.

### Selection analysis

Rare variants are predominantly affected by non-adaptive factors, whereas selection mainly affects variants with higher population frequencies^16–18^. Based on this principle, we assessed selection trends for each gene. First, we obtained all missense and synonymous variants with an average depth (DP) > 5 and AN > 150. Then, the number of missense and synonymous variants for each gene was calculated for two AF ranges: less than 0.01, representing rare to low-frequency variants, and greater than 0.03, representing low to common polymorphism. Then, the per-gene missense to synonymous ratio (M/S) was calculated for each range, as described previously^16^. Lastly, we calculated the M/S_<0.01_ to Ms/S_>0.03_ ratio for each gene as an indicator for selection direction. Thus, if functional substitutions are adaptive in nature, then the M/S_<0.01_ to M/S_>0.03_ ratio is expected to be ≫ 1, and vice versa for purifying selection. Top 100 genes with M/S_<0.01_ to M/S_>0.03_, all with ratios > 2, were further assessed for their functional contribution by enrichment analysis using GeneAnalytics^19^.

## Data Records

The compressed ExAgBov database file can be downloaded from the Mendeley Data repository^13^. The database has three components. The first seven columns are in the VCF format, followed by four columns denoting the allele count (AC), AN, AF, and inbreeding coefficient. A description of the VCF file format can be found at https://gatk.broadinstitute.org/. The second section includes all the VEP annotations of the variant. A description of the VEP annotations format can be found at https://www.ensembl.org/info/docs/tools/vep/script/index.html. The last three columns provide the variant sequencing depth information. The following files can be downloaded from the ExAgBov data repository^13^. The filtered database file includes variants that pass the quality filtration for the read quality and depth of coverage in the BovExAg.DB.TSV + DP.filtered.gz file. A list of all the SRA sample accessions and their breed affiliations is provided in the SRA.ID + breed.csv file. The complete SRA metadata is reported in the ExAgBov.SRA.metadata.txt file. The sequencing depth of each variant in each WES sample can be found in the ExAgBov.DP.all-var.csv.gz file. The fastq files of all samples can be retrieved from the SRA repository (https://www.ncbi.nlm.nih.gov/sra) using the identifiers denoted in the SRA.ID + breed.csv file.

## Technical Validation

**Fig. 1:**
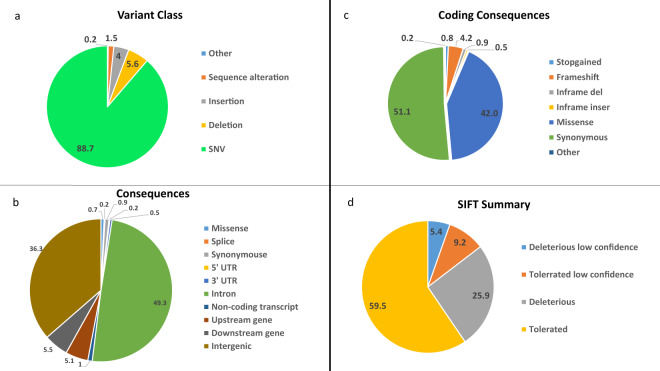
ExAgBov descriptive statistics. Graphs showing the distribution of variant class (**a**), variant consequences (**b**), variant coding consequences (**c**), and functional prediction (**d**). About 2% of all variants are located within exonic regions and about 1% are expected to cause alteration in the protein sequence (e.g., missense and splice variation).

**Fig. 2:**
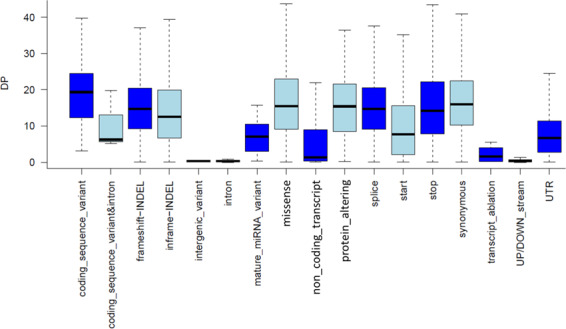
Average depth distribution of ExAgBov variants as a function of their consequence category. The sequencing depth (DP) of all variants in all samples was obtained (see additional files at 10.17632/m3p9m9vc4g.3^13^), and the per-variant average DP (Y-axis) was calculated and summarized for each variant category (X-axis).

**Fig. 3:**
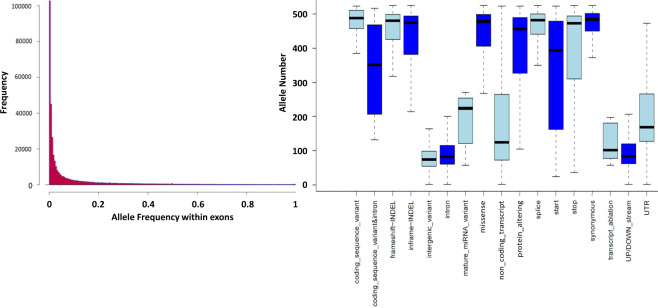
Allele number distribution for all variants and allele frequency distribution for all exonic variants. Right panel: AN distribution of all variants as a function of the variant category (X-axis). Exonic variants were highly divergent from intergenic and intronic variants, which were covered in less than half of the samples. Therefore, AF distribution (left panel) is shown for variants located within exon sequences (i.e., the “targeted” genome). Most exonic variants were rare to low frequency (AF < 0.03; left panel) and were covered in most samples (AN > 450). Y-axis denotes the histogram frequencies.

The ExAgBov database contains more than 20 million short variants, most of which were SNVs (Fig. 1a). Although WES was designed to selectively capture exonic regions, most of the identified variation was found in non-exonic regions, mainly in intronic and intergenic regions (Fig. 1b). About 400,000 variations (SNVs and INDELs) were located within exonic regions. Nearly half of them predicted protein alteration (e.g., missense and splice variants, Fig. 1b,c). 30–40% of the missense variants were predicted deleterious (Fig. 1d). Analysis of the average depth distribution of all variants as a function of their consequence showed that most exonic variants had an average depth > 20 (Fig. 2) and were covered in all samples, as reflected in the AN distribution (Fig. 3). By contrast, intergenic and intronic variants had a very low sequence coverage. They were covered in less than half of the samples (Figs. 2 and 3). This is possibly due to significant differences in the overall sample coverage, as shown in Figure S2. Thus, the reliability of the AF of the intergenic variants is low and should be carefully assessed for each variant. Alternatively, we produced an additional filtered database file that retained only those variants that pass our qualities criteria as denoted in the methods section. The filtered database includes 2.7 million variants, and the variants’ consequences distribution is represented in figure S1. Most exonic variants were rare, with an AF lower than 0.01 (Fig. 3). The high number of variants and the AF distribution might be unexpected given the recent reduction in effective population size; however, similar results were reported previously^3^.

**Fig. 4:**
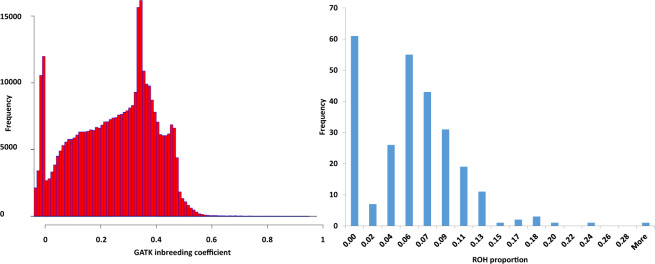
Distribution of inbreeding coefficient and ROH in all exon variants. The inbreeding coefficient (X-axis; left panel) was calculated for all exonic variants. Most variants had low-to-intermediate positive inbreeding coefficient values, indicating less heterozygosity than expected. This is likely due to the relatively high inbreeding in commercial herds^20,22,25^, as reflected by ROH distribution (X-axis; right panel) among the ExAgBov samples.

Because the ExAgBov database contains information obtained from an uncontrolled population, we performed a set of analyses to evaluate the population structure and additional factors that could bias the AF. First, we calculated the per-variant GATK inbreeding coefficient. This measurement indicates the degree of deviation from the Hardy-Weinberg equilibrium, thereby exposing biases resulting from the structure of the sampled population. The inbreeding coefficient distribution of exonic variants showed mostly low to intermediate positive values (Fig. 4). This could result from the presence of closely related animals in the sampled population or the relatively high inbreeding among sampled individuals, as has been observed in modern commercial cattle herds^20–22^. To assess the contribution of inbreeding, we calculated the per-sample ROH proportion. The results showed relatively high levels of inbreeding for many animals, as the ROH of several animals exceeded 10% (Fig. 4). Such ROH levels are similar to those previously observed in commercial herds, resulting from the small effective population size and the intensive genetic and genomic selection strategies^20–22^. This observation can explain, at least partially, the high inbreeding coefficient observed for some of the variants.

**Fig. 5:**
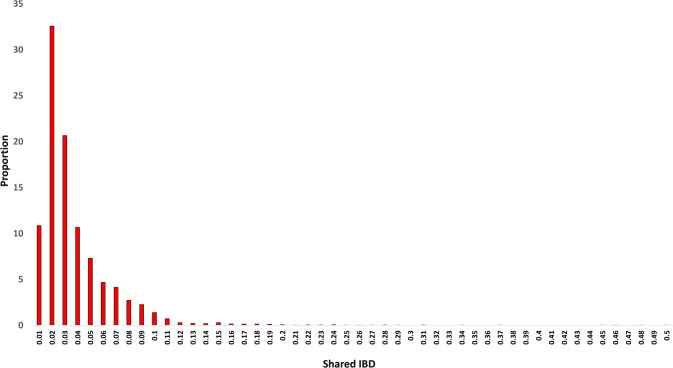
Distribution of shared IBD segments among all bovines WES samples. The proportion of shared IBD (X-axis) of all ExAgBov sample pairs is presented. The frequencies of shared IBD (Y-axis) for most pairs indicate non- or remote kinship.

Next, we assessed kinship levels among ExAgBov samples by computing the shared IBD between all pairs (Fig. 5). Overall, most of the bovine samples shared none or small IBD genomic segments with each other. We detected a shared IBD pattern for seven bovine pairs that can be inferred as a full sibling, and 10–20 pairs might be suspected as second-degree relatives. All other pairs of samples were non- or remotely related.

**Fig. 6:**
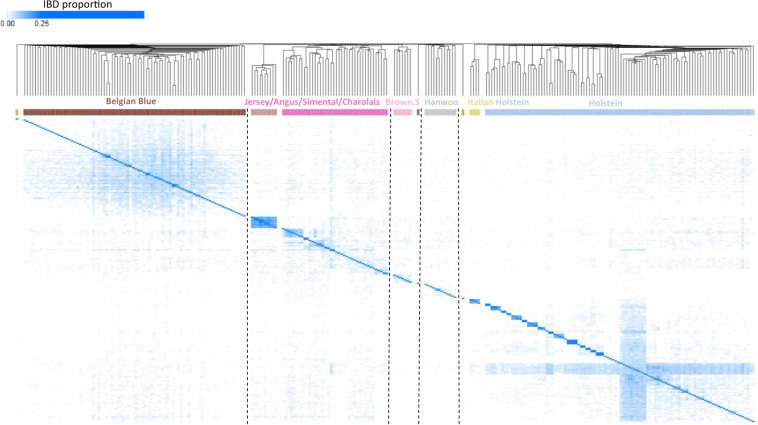
ExAgBov relationship matrix. The matrix of all shared IBD segments between all pairs of WES samples underwent hierarchical clustering. Breed affiliation was either indicated in the SRA metadata (see 10.17632/m3p9m9vc4g.3^13^) or inferred from the existing data. X and Y-axes are the bovine WES samples. The dashed lines denote clusters of bovine samples and their assumed breed affiliation. The color-coded IBD proportion indicates the kinship degree of each pair. Seven pairs are likely siblings.

**Fig. 7:**
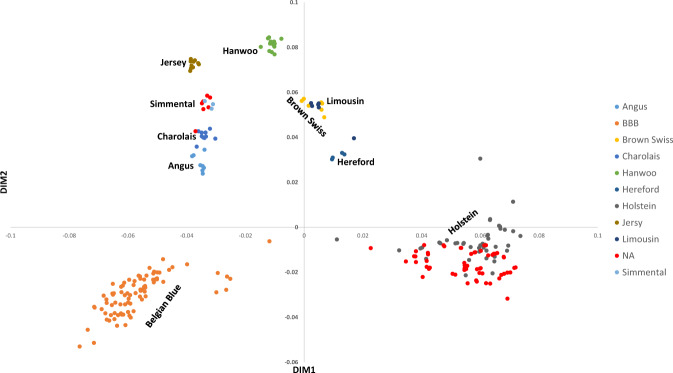
Multidimensional scaling plot of the ExAgBov population structure. MDS analysis was performed on the computed matrix of the genome-wide IBS pairwise distances, and the projection of the two first dimensions is shown. The breeds were obtained from the metadata (see 10.17632/m3p9m9vc4g.3^13^) or inferred from the existing data. NA, not applicable; BBB, Belgian Blue Beef. X and Y-axes represent the first and the second MDS dimension values, respectively.

The clustered kinship matrix revealed 4–7 major clusters of bovine samples that were more related to one another (Fig. 6). We therefore used information about breed affiliation, which was available for some samples, to infer the affiliation of other samples within the same cluster (Fig. 6). Next, we performed MDS analysis to uncover the population structure of the ExAgBov database (Fig. 7). Combining the MDS analysis with the data from the clustered kinship matrix, we inferred the breeds composing the ExAgBov database. We found that ExAgBov mainly comprises Holstein bulls from several herds as well as Belgian Blue (BBB). In addition, it includes several beef breeds, like Angus and Simental, and dairy breeds like Brown Swiss.

**Fig. 8:**
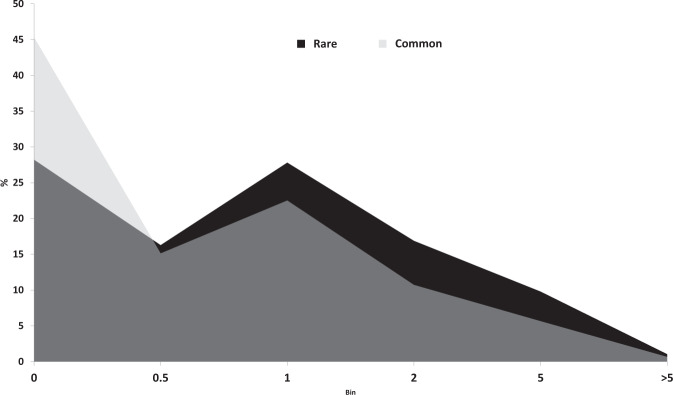
Distribution of M/S ratios. The per-gene missense to synonymous ratio (M/S, X-axis) was calculated as a function of the AF range. The distribution of the M/S for rare variants (AF < 0.01) shows a shift to the right (higher M/S), indicating an overall purifying selection.

Because cattle herds underwent intensive genetic selection as well as continuous adaptation to environmental constraints^23^, we used the ExAgBov data to preliminary assess the per-gene selection trends. For that, we computed the ratio between missense variants, which are likely functional substitutions, and synonymous variants, likely neutral substitutions (M/S). We then compared the M/S ratios of rare to low-frequency variants (AF < 0.01; M/S_<0.01_) to those of moderate-to-common polymorphism (AF > 0.03; M/S_>0.03_). We found that the M/S ratio of common variants tended to be lower than that of rare variants (M/S_>0.03 < _M/S_<0.01_; Fig. 8), indicating an overall purifying selection. This was expected, as likely functional variants tend to be purged from the population more than neutral variants. We then calculated the rare-to-common M/S ratio for each gene to identify genes that might have undergone adaptive selection. Higher ratios indicate less purifying selection and likely adaptive selection. To assess the functional involvement of genes with a ratio indicating likely adaptive selection, we performed enrichment analysis on the 100 genes with the highest M/S_<0.03_ to M/S_<0.01_ ratios (Supplementary Table 1). We found that these genes were associated with protein metabolism, protein synthesis, immune response, and host-pathogen interaction (Supplementary Table 2). Adaptive selection is well established in genes affiliated with host-pathogen interaction and the immune system and has been demonstrated in several species populations, including cattle^24^. The seemingly rapid evolution of genes associated with protein synthesis and metabolism might result from the intensive genetic selection for productive traits^23^.

## Usage Notes

The ExAgBov database can be used directly to obtain variations in different genomic regions or variants associated with a specific gene. This can be done by extracting relevant lines using simple command lines or scripts. For instance, Linux users can custom the grep command with the gene name and the term “missense” to obtain all missense variants within a certain gene (e.g., zcat BovEx.DB.TSV.gz| grep –e “NDUFC2” | grep –e “missense”: will retrieve all missense variants in the gene NDUFC2). The main usage of the ExAgBov database is for comprehensive annotation of NGS-driven variants, like in WES and WGS (Table 1). This is done by customizing a script that adds the desired information to identified variants of NGS analysis whenever recorded within the ExAgBov database. A more suitable way to implement the ExAgBov data when comprehensively annotating NGS-driven variants is by formatting and adding the AN, AF, average-DP, and inbreeding coefficient information to the Ensembl VEP pipeline. VEP enables the integration of custom annotation from simple format files into the annotated variant call file by using the --custom flag (see https://m.ensembl.org/info/docs/tools/vep/script/vep_custom.html). Lastly, the ExAgBov allows to accurate the reference genome data for variants that exceed 50% AF and, thereby, better representation of the bovine consensus sequence.

**Table 1:** Applying the ExAgBov database for WGS analysis.

CHR.^1^	POS	REF	ALT	Consequence	Gene	AF	AN	IC
4	25851940	C	T	missense	ENSBTAG00000007746	0.025	522	0.38
8	94696012	G	A	splice_region	ENSBTAG00000020661	0.035	520	−0.028
10	1191682	C	T	missense	ENSBTAG00000018852	0.019	522	0.26
8	80873738	C	T	splice_region	ENSBTAG00000000738	0.089	518	0.17
1	157758838	C	T	missense	ENSBTAG00000051758	0.045	286	0.06
10	26636185	T	A	missense	ENSBTAG00000012317	0.021	288	−0.019
10	26648481	G	C	missense	ENSBTAG00000037452	0.06	250	0.07
4	112208828	G	A	splice_region	ENSBTAG00000017143	0.069	494	−0.02
8	89125920	TG	T	frameshift	ENSBTAG00000011374	0.011	522	0.259
4	48998296	G	C	missense	ENSBTAG00000011412	0.004	494	0.436
28	9182425	A	G	missense	ENSBTAG00000013544	0.055	492	0.399
10	19988964	A	G	missense	ENSBTAG00000004990	0.063	504	0.17
27	7686074	G	A	missense	ENSBTAG00000014135	0.032	474	0.13

^1^CHR., chromosome; POS, position; REF, reference allele; ALT, alternative allele; AF, ExAgBov allele frequency; AN, ExAgBov allele number; IC, inbreeding coefficient.

Annotation of variants identified in WGS analysis in a study of Israel Holstein using the ExAgBov database (unpublished results). The AF, AN, and the inbreeding coefficient (IC) were retrieved for each variant for downstream filtering.

## Supplementary information

Supplementary Table 2

Supplementary Figure 1

Supplementary Figure 2

Supplementary Table 1

## Data Availability

The full pipeline is available at https://github.com/morangershoni/BovEx. All software used in this study are freely available: SRA Toolkit: https://github.com/ncbi/sra-tools. BWA: https://github.com/lh3/bwa. Picard Tools: https://broadinstitute.github.io/picard/. GATK software: https://github.com/broadinstitute/gatk/releases. VEP: https://m.ensembl.org/info/docs/tools/vep/script/vep_download.html. PLINK: https://zzz.bwh.harvard.edu/plink/download.shtml. KING: https://www.kingrelatedness.com/Download.shtml.
