# Supplementary material for: ExAgBov: A public database of annotated variations from hundreds of bovine whole-exome sequencing samples
Source: Sci Data. 2022 Aug 2;9:469. doi: 10.1038/s41597-022-01597-8 (PMC9345876; doi:10.1038/s41597-022-01597-8)
Supplement: Supplementary file 3 — Supplementary Figure 2 [file 41597_2022_1597_MOESM3_ESM.pdf]

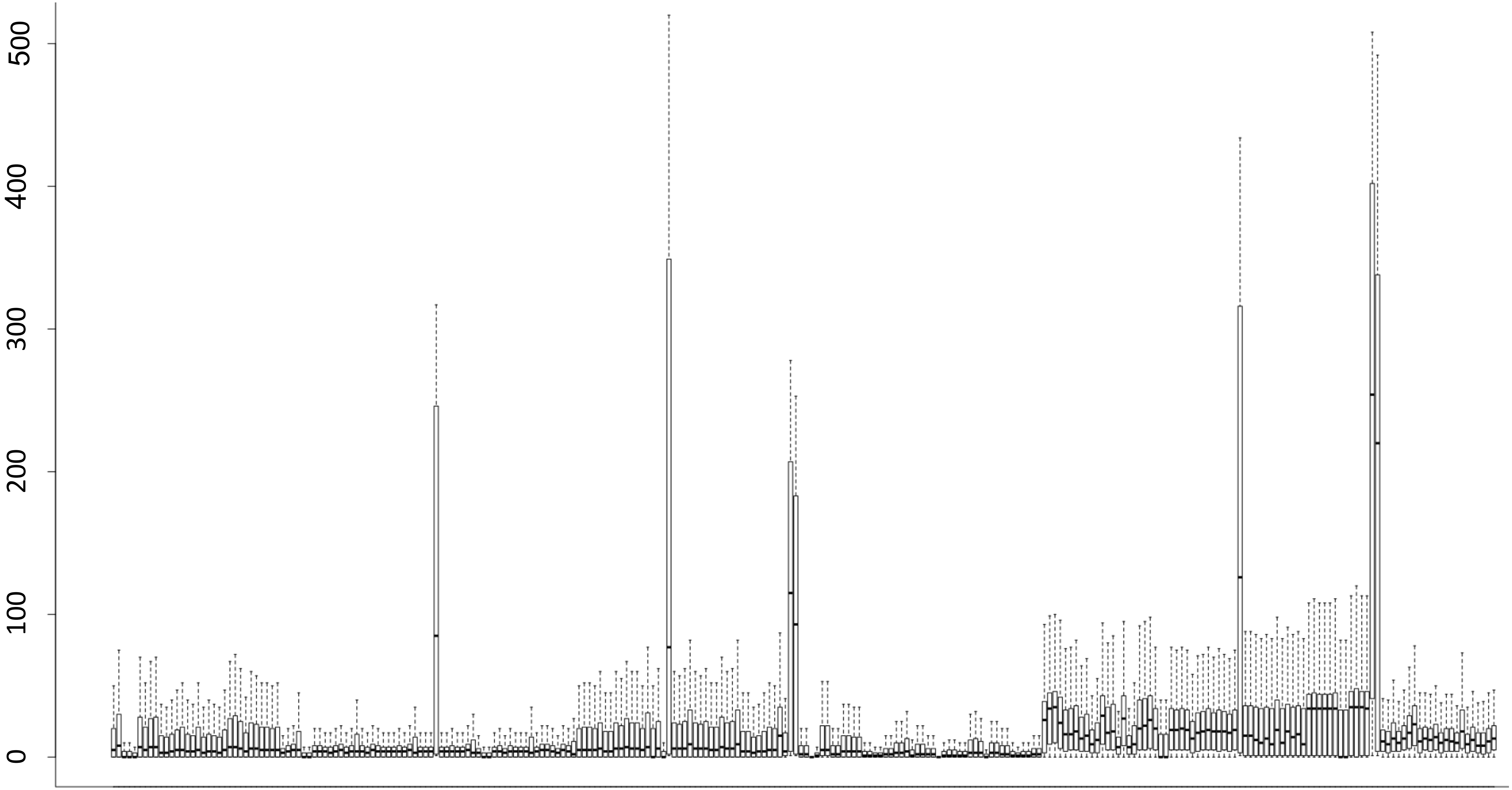

Supplementary Figure 1. boxplot describing the distribution of the per-sample depth of coverage
